# Supplementary material for: End Sequence Analysis Toolkit (ESAT) expands the extractable information from single-cell RNA-seq data
Source: Genome Res. 2016 Oct;26(10):1397–410. doi: 10.1101/gr.207902.116 (PMC5052061; doi:10.1101/gr.207902.116)
Supplement: Supplemental Material [file supp_gr.207902.116_Supp_Materials.tar.gz › ESAT-esat_v0.1/doc/html/classbroad_1_1pda_1_1gene_1_1_neighbors_null_model.html]

ESAT: broad.pda.gene.NeighborsNullModel Class Reference


ESAT


- Main Page
- Related Pages
- Packages
- Classes
- Files

- Class List
- Class Index
- Class Hierarchy
- Class Members

All Classes Namespaces Files Functions Variables Enumerator Pages

- broad
- pda
- gene
- NeighborsNullModel

Classes |
Public Member Functions |
List of all members

broad.pda.gene.NeighborsNullModel Class Reference

|  |  |
| --- | --- |
| Classes | |
| class | **chrSizeNode** |
|  | |
| class | **NeighborsBin** |
|  | |

|  |  |
| --- | --- |
| Public Member Functions | |
|  | NeighborsNullModel () |
|  | |
|  | NeighborsNullModel (String setname, String refname, int binsize, int permnum) throws IOException |
|  | |
|  | NeighborsNullModel (BEDFileParser setname, BEDFileParser refname, int binsize, int permnum) throws IOException |
|  | |
| void | makeBinnedIntervalModel (boolean UseCustomBins) |
|  | |
| void | makeNaiveRandModel (String chrSizeF) throws IOException |
|  | |
| void | makeDartThrowingModel () |
|  | |
| ArrayList< GeneWithIsoforms[]> | getGeneRandNeighbors (Gene gene) |
|  | |
| void | printRandNeighborsInfo () |
|  | |
| int | getPermNum () |
|  | |
| HashMap< Gene, ArrayList  < GeneWithIsoforms[]> > | getGeneRandNeighbors () |
|  | |
| Collection< Gene > | getUniqueNeighbors () |
|  | |

## Constructor & Destructor Documentation

|  |  |  |  |  |
| --- | --- | --- | --- | --- |
| broad.pda.gene.NeighborsNullModel.NeighborsNullModel | ( |  | ) |  |

|  |  |  |  |
| --- | --- | --- | --- |
| broad.pda.gene.NeighborsNullModel.NeighborsNullModel | ( | String | *setname*, |
|  |  | String | *refname*, |
|  |  | int | *binsize*, |
|  |  | int | *permnum* |
|  | ) |  | throws IOException |

|  |  |  |  |
| --- | --- | --- | --- |
| broad.pda.gene.NeighborsNullModel.NeighborsNullModel | ( | BEDFileParser | *setname*, |
|  |  | BEDFileParser | *refname*, |
|  |  | int | *binsize*, |
|  |  | int | *permnum* |
|  | ) |  | throws IOException |

## Member Function Documentation

|  |  |  |  |  |  |
| --- | --- | --- | --- | --- | --- |
| ArrayList<GeneWithIsoforms[]> broad.pda.gene.NeighborsNullModel.getGeneRandNeighbors | ( | Gene | *gene* | ) |  |

|  |  |  |  |  |
| --- | --- | --- | --- | --- |
| HashMap<Gene, ArrayList<GeneWithIsoforms[]> > broad.pda.gene.NeighborsNullModel.getGeneRandNeighbors | ( |  | ) |  |

|  |  |  |  |  |
| --- | --- | --- | --- | --- |
| int broad.pda.gene.NeighborsNullModel.getPermNum | ( |  | ) |  |

|  |  |  |  |  |
| --- | --- | --- | --- | --- |
| Collection<Gene> broad.pda.gene.NeighborsNullModel.getUniqueNeighbors | ( |  | ) |  |

|  |  |  |  |  |  |
| --- | --- | --- | --- | --- | --- |
| void broad.pda.gene.NeighborsNullModel.makeBinnedIntervalModel | ( | boolean | *UseCustomBins* | ) |  |

Here is the call graph for this function:

|  |  |  |  |  |
| --- | --- | --- | --- | --- |
| void broad.pda.gene.NeighborsNullModel.makeDartThrowingModel | ( |  | ) |  |

|  |  |  |  |  |  |
| --- | --- | --- | --- | --- | --- |
| void broad.pda.gene.NeighborsNullModel.makeNaiveRandModel | ( | String | *chrSizeF* | ) | throws IOException |

Here is the call graph for this function:

|  |  |  |  |  |
| --- | --- | --- | --- | --- |
| void broad.pda.gene.NeighborsNullModel.printRandNeighborsInfo | ( |  | ) |  |

---

The documentation for this class was generated from the following file:

- java/broad/pda/gene/NeighborsNullModel.java


---

Generated on Fri Aug 15 2014 15:11:00 for ESAT by  

 1.8.7
